# UV Treatment of Flexible Copper Nanowire Mesh Films for Transparent Conductor Applications

**DOI:** 10.1186/s11671-017-2343-y

**Published:** 2017-10-30

**Authors:** Quentin Lonne, Jose Endrino, Zhaorong Huang

**Affiliations:** Surface Engineering & Nanotechnology Institute, Cranfield University, College Road, Cranfield, Bedfordshire, MK43 0AL UK

**Keywords:** Copper nanowires, Transparent conductive electrodes, Ultra-violet treatment

## Abstract

Copper nanowires have the potential to reach and even exceed the indium tin oxide performances as flexible transparent conductive electrodes. However, for a large-scale production, they need to be fabricated in a high-speed, low-cost way, without degrading the flexible substrate. One of the major bottlenecks resides in the post-treatment used to remove organic residues from the surface of the nanowires after forming the transparent electrode, which is necessary to obtain high optoelectronic performances. Here, we propose an ultra-violet irradiation and a subsequent acetic acid bath as an easy, scalable, fast post-treatment. After only 2 min of ultra-violet treatment, followed by 10 min of acid bath, an *R*s of 42 Ω sq^−1^ and a *T*_550 nm_ of 87% were measured. Besides, copper nanowire electrodes maintained their high transparency in the range 750–2500 nm, which makes them good candidates for applications such as infrared solar cells.

## Background

The use of transparent conductive electrodes (TCEs) is essential in many everyday devices such as touch screens, displays, solar cells and light-emitting diodes [1–5]. Requirements for that type of component are outstanding optoelectronic properties fitting the desired applications and a low-cost, large-scale production method. The TCE transparency at a wavelength of 550 nm, *T*_550 nm_, is typically ca. 90%. Their sheet resistance, *R*s, can vary from ≤ 20 Ω sq^−1^ for solar cells to ≥ 100 Ω sq^−1^ for capacitive touch screens [1–5].

Currently, indium tin oxide (ITO) is the most common material for TCEs but it presents several drawbacks. It is expensive due to the indium scarcity and the slow physical vapour deposition process used. Moreover, it is brittle [1–5], which hinders its use for organic, flexible or bendable applications. Indeed, it forms microcracks after a few bending cycles, which considerably reduces the TCE electrical conductivity [6–10]. To address these issues, researchers have focused on various alternative materials such as poly(3,4-ethylenedioxythiophene) poly(styrenesulfonate) [11, 12], graphene [13, 14], carbon nanotubes [15, 16], Ag nanowires (NWs) [17–19] and Cu NWs [3, 5]. The latter is one of the most promising materials due to Cu abundance, low cost and high electronic conductivity [3, 5]. Besides, Cu NWs can be fabricated through a low-cost, large-scale, wet chemical synthesis [20–22] and deposited with a low-cost, high-speed, roll-to-roll (or reel-to-reel, R2R) process [6, 9]. Finally, their high flexibility allows the TCE to maintain stable performances even after 1000 bending cycles [7, 8, 10, 23, 24].

The Cu NW chemical synthesis involves a capping agent, generally an alkylamine such as oleylamine (OM) [10, 22, 24–27], octadecylamine [28, 29], hexadecylamine [8, 20, 30, 31] or ethylenediamine [7, 21, 23, 32], which makes Cu nanoparticles (NPs) grow anisotropically. The NW aspect ratio (length/diameter) is of the utmost importance because the higher it gets, the lower the area fraction covered by the NWs needs to be to obtain a percolated network and the more transparent the TCE is [33–36]. However, those capping agents leave residues on the surface of the NWs, even after an extensive washing in various solvents. Besides, prior to the TCE formation, the NWs are often put in suspension into a nano-ink using a dispersing agent such as polyvinylpyrrolidone (PVP) [22, 23, 26, 30] or nitrocellulose [7, 32]. All those organic residues hinder the NW good contact in the mesh film, and hence, significantly decrease the TCE conductivity. Indeed, Mutiso et al. demonstrated that the sheet resistance of a NW TCE is almost equivalent to the contact resistance between the NWs [36].

Consequently, a post-treatment is necessary to remove organic residues after forming a Cu NW TCE. It is generally a high-temperature treatment under a vacuum [24, 25], inert [22], reducing (pure H_2_) [7] or forming (5% H_2_–95% inert gas) [26, 27] atmosphere. This avoids Cu oxidation while removing organic residues and fusing the NW junctions. However, this suits neither a high-rate, low-cost production nor a low-fusion temperature, flexible, polymer substrate. Therefore, alternative post-treatments have been tested and have given very promising results. Treatments using lactic [8], hydrochloric [30], propionic [27] or acetic [10, 29] acid, for instance, proved to be very efficient to remove organic residues from the surface of Cu NWs without damaging the polymer substrates. After an acetic acid treatment, Mayouse et al. obtained poly-ethylenenaphthalate-supported TCEs with *R*s values of 9 and 55 Ω sq^−1^ for a respective *T*_550 nm_ of 88 and 94% [29]. Using the same acid, Wang et al. developed TCEs on polyethylene terephthalate (PET) substrates with an *R*s of 30 and 60 Ω sq^−1^ for respective *T*_550 nm_ values of 83 and 90% [10]. Besides, photonic sintering using xenon flash lamp pulses allowed to fuse the NW junctions while removing undesired organics in ambient air in a few milliseconds [31, 37]. Ding et al. reported 23 Ω sq^−1^ for *T*_550 nm_ = 82% [37]. Mallikarjuna et al. obtained an *R*s of 110 and 170 Ω sq^−1^ for a *T*_550 nm_ of 90 and 95%, respectively [31]. Hence, although photonic sintering seems very promising, further efforts must be made to obtain *R*s < 100 Ω sq^−1^ with *T*_550 nm_ ≥ 90%.

In this work, we synthesised high aspect ratio Cu NWs using OM as a solvent, capping and reducing agent, and a nickel (II) species as a catalyst. The NWs were then dispersed in an ink and coated on flexible PET substrates to form TCEs. A post-treatment was necessary to obtain both high conductivity (42 Ω sq^−1^) and transparency (87% in the visible range). It involved an irradiation under an ultra-violet (UV) lamp, followed by an acetic acid bath, which are both compatible with a R2R process [6, 9, 38, 39]. The UV-treated Cu NW TCEs were compared to conventionally, thermally treated Cu NW TCEs and to commercial ITO.

## Experimental Section

Copper (II) chloride dihydrate (CuCl_2_·2H_2_O, ≥ 95.0% pure), nickel (II) acetate tetrahydrate (Ni(C_2_H_3_O_2_)_2_·4H_2_O, ≥ 99.0% pure), OM (C_18_H_37_N, 70% pure), anhydrous hexane (C_6_H_14_, 95.0% pure), acetic acid (C_2_H_4_O_2_, ≥ 99% pure), ethyl acetate (C_4_H_8_O_2_, ≥ 99.7% pure) and PVP ((C_6_H_9_NO)_*n*_, 10,000 g mol^−1^) were purchased from Sigma Aldrich UK. Isopropyl alcohol (IPA, (C_3_H_8_O, ≥ 99.5% pure), PET substrates ((C_10_H_8_O_4_)_*n*_, 125 ± 25 μm thick) and a glass-supported ITO TCE were purchased from Fisher Scientific UK, Goodfellow UK and Optics Balzers, Liechtenstein, respectively. All chemicals were used as received.

The Cu NW synthesis process was based on a catalytic method previously reported by Guo et al. [25]. 0.4092 g (2.4 mmol) of CuCl_2_·2H_2_O, 0.2986 g (12 mmol) of catalytic Ni(C_2_H_3_O_2_)_2_·4H_2_O, 25 mL of OM and a magnetic stirrer were added in a 50-mL round bottom flask. The flask was placed in an oil bath on a magnetic stirring hotplate (model 3810000 RCT Basic IKAMAG, IKA) and connected to a reflux column with a top, in-line, oil bubbler. The solution was first heated at 90 °C for 30 min under a vigorous 800-rpm stirring and a constant N_2_ flow to remove O_2_(g) and water traces. At that stage, the solution was blue. Then, the temperature was increased to 190 °C to reduce Cu^2+^ ions and form Cu^0^ seeds, and the colour of the solution progressively became red. After 30 min, the stirring was stopped and the solution kept at 190 °C under N_2_ for 16 h to make the Cu NWs grow from the seeds. Finally, the heating was stopped and the solution allowed to cool down naturally.

The solution was transferred to a 50-mL vial and washed successively with hexane, IPA, acetic acid and IPA again. In each solvent, the Cu NWs were vortexed 2 min in manual mode (model Topmix FB15024, Fisher Scientific) and then centrifuged at 4000 rpm (model AccuSpin 400, Fisher Scientific). Centrifugation lasted 20 min in hexane and 2 min in the other solvents. Finally, the Cu NWs were incorporated into an ink composed of 26 vol% of ethyl acetate and 74 vol% of IPA containing 0.5 wt% of PVP. The Cu NW ink was vortexed at 10 Hz for 30 min before storage. The Cu NW concentration in the ink was either 10 or 20 mg mL^−1^.

Before the coatings were carried out, the Cu NW ink was vortexed once more at 10 Hz for 5 min. To coat a 10 × 10-cm^2^ PET substrate, 100 μL of ink were taken with a micro-pipette and put on the substrate to form a straight liquid line parallel to the upper edge. The ink was immediately and quickly spread over the PET substrate with a Meyer rod (N°4 from Dyne Testing UK, giving a ca. 10.2-μm thick wet film). All solvents were evaporated after a few seconds at room temperature.

Two different post-treatments were implemented on the as-obtained Cu NW TCEs to remove organic residues (OM and PVP). Some TCEs underwent a thermal treatment at 200, 210, 220, 230, 240 or 250 °C during 1 h under N_2_ in a tubular oven (model MTF 10/25/130, Carbolite). The other ones underwent a UV irradiation in ambient air for 2, 4 or 6 min with a 430-W lamp (model UVASPOT 400/T, Honle). The lamp was equipped with a mercury vapour bulb (H type), and the distance between the bulb and the samples was 30 cm. After either thermal or UV treatments, TCEs were dipped into pure acetic acid for 10 min to further remove organics and possible oxide traces.

The structure of the Cu NWs was determined using an X-ray diffractometer (XRD, model D5005, Siemens) with a Bragg Brentano configuration chamber, a Cu anticathode (*K*_α_ = 0.154184 nm) and a back monochromator. The X-ray patterns were indexed with a DIFFRAC.SUITE EVA software (Bruker AXS) containing the JCPDS files database. The microstructure and composition were characterised using a scanning electron microscope equipped with a field emission gun (FEG-SEM, model XL30 SFEG, Philips) and an in-situ energy dispersive spectrometer (EDS, Oxford Instruments-AZTEC). The TCE sheet resistance and transmittance were measured using the four-point probe technique (model 3007 A, Kulicke & Soffa) and a UV-Vis/NIR (near infrared) spectrophotometer (model V-670, JASCO), respectively.

The manufacturing parameters of various TCEs, as well as their ID, *R*s and transparency, are summarised in Table 1. However, the TCEs which had an *R*s so high that it could not be measured by the four-point probe technique are not included in this table and have no specific ID.

**Table 1:** ID, sheet resistance (*R*s) and transmittance values (*T*_550 nm_, *T*_350–750 nm_ and *T*_750–2500 nm_) of various TCEs

Sample ID	Ink concentration/mg mL^−1^	Post-treatment	*R*s/Ω sq^−1^	*T*_550 nm_	*T*_350–750 nm_	*T*_750–2500 nm_
ITO/ref	n.a.	n.a.	10	90	84	50
#1	20	220 °C/1 h	25	61	61	65
#2	20	230 °C/1 h	743	46	45	57
#3	10	UV/2 min	42	87	87	89
#4	10	UV/4 min	103	89	89	91
#5	20	UV/2 min	31	67	67	68
#6	20	UV/4 min	49	70	71	74
#7	20	UV/6 min	236	73	73	76

The values are given for a commercial, glass-supported, ITO TCE and for either thermally or UV-treated, PET-supported Cu NW TCEs. *T*_550 nm_ corresponds to the transparency at a wavelength of 500 nm, and *T*_350–750 nm_ and *T*_750–2500 nm_, to the average transparency in the ranges 350–750 nm and 750–2500 nm, respectively

*n.a.* not applicable

## Results and Discussion

**Fig. 1:**
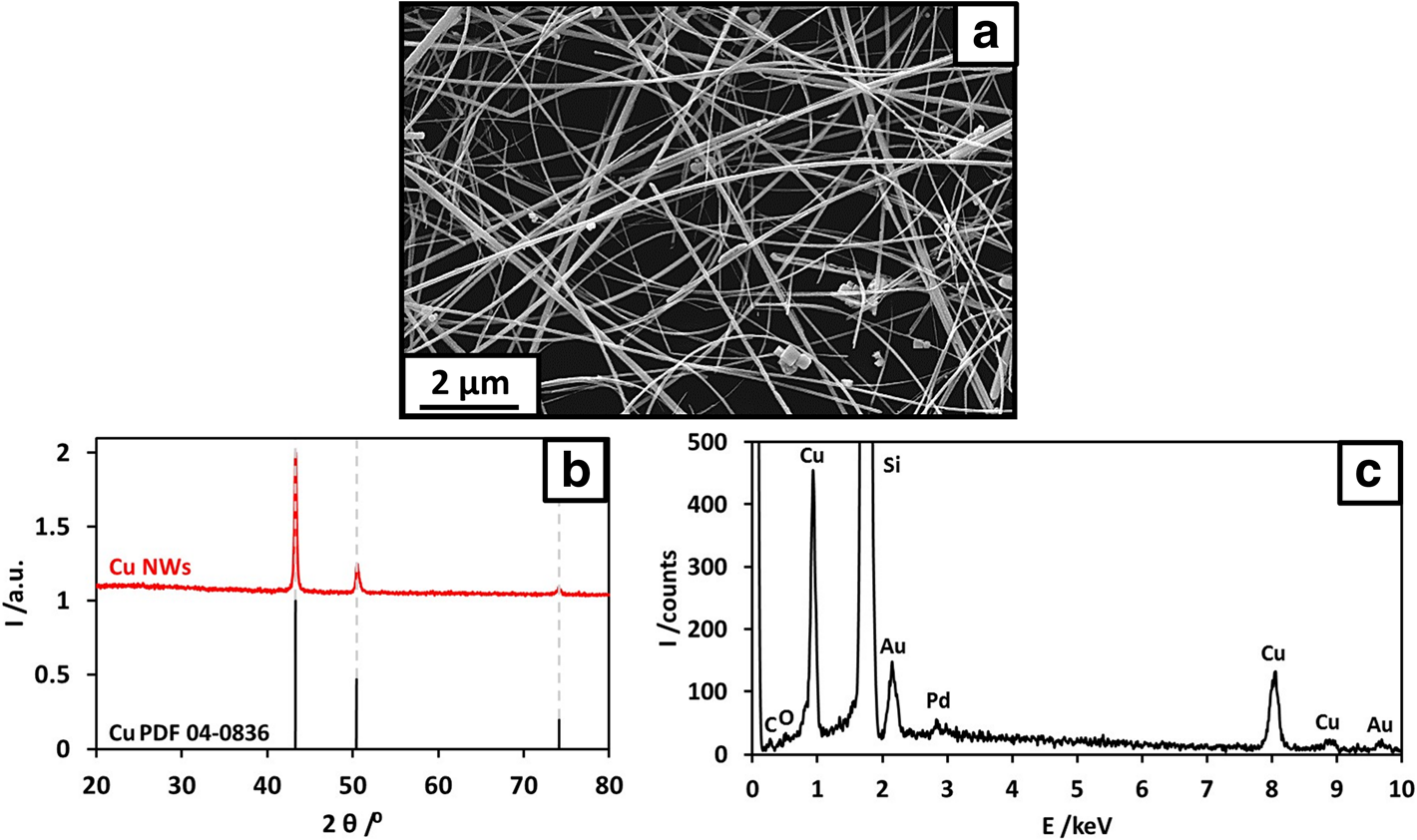
As-synthesised Cu NWs before being incorporated in the nano-ink. **a** SEM image showing the high aspect ratio (~ 1000) of the Cu NWs and a few cubic Cu NPs. **b** XRD pattern. **c** EDS spectrum showing the high purity of the washed Cu NWs

The synthesised, washed Cu NWs exhibited a high aspect ratio of ca. 1000 (average length and diameter of 70 μm and 70 nm, respectively) with very few cubic NPs, as seen in Fig. 1a. The presence of the latter suggests slow Ni^2+^ reduction kinetics [3]. The XRD pattern of Fig. 1b proves that the NWs were made of Cu with a face-centred cubic structure Fm3m (in agreement with the PDF file 04-0836), without any secondary phase detected within the limit of the apparatus (ca. 5 wt%). In particular, there is no diffraction peak corresponding to a copper oxide or a Ni-containing phase. The high purity of the Cu NWs is further confirmed by the EDS spectrum in Fig. 1c. The traces of carbon and oxygen were attributed to non-crystalline OM residues as no other phase than pure Cu was revealed by XRD, and it is well-known that it is very difficult to remove all the OM without a post-treatment [10, 25, 27]. No trace of Ni was found within the detection limit of the EDS (ca. 0.1 wt%), confirming that its role during the synthesis was mainly catalytic as previously described [25, 26]. The silicon corresponds to the wafer supporting the NWs during the EDS analysis and the gold and palladium, to the metallic nano-coating used to improve the sample conductivity and therefore the analysis quality.

**Fig. 2:**
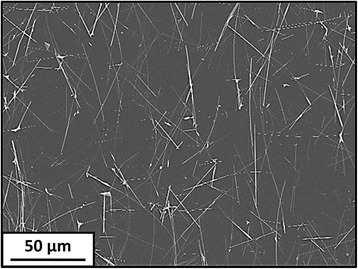
Low-magnification SEM image of the UV-treated TCE #3 (2 min) on a PET substrate: it shows Meyer rod-coated Cu NWs forming a well-dispersed, percolated network

After forming the TCEs using a Meyer rod, either thermal or UV post-treatments were used to remove OM and PVP residues from the Cu NW surface and try to fuse them together. Figure 2 shows the surface of the TCE #3 where the NWs form a percolated network, which is necessary for the TCE to be conductive through its entire area. The NWs appear very well dispersed, without any aggregate or bundle that would decrease the TCE transparency. This confirms that the Meyer rod coating is an easy, fast and efficient process to obtain large-area, well-dispersed, percolated NW TCEs [2, 7, 32].

**Fig. 3:**
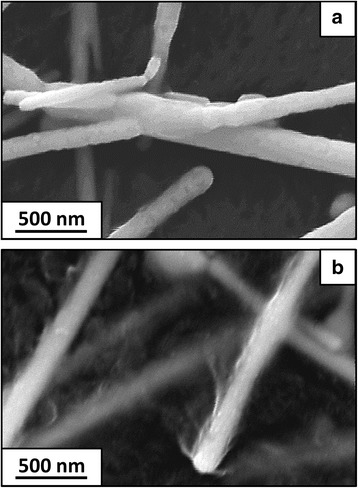
SEM images of thermally treated, PET-supported TCEs. **a** Fused (TCE #1, 220 °C/1 h). **b** PET-encapsulated (TCE #2, 230 °C/1 h) Cu NWs

Figure 3 shows thermally treated TCEs, with fused (Fig. 3a) and PET-encapsulated (Fig. 3b) Cu NWs. Indeed, during the thermal treatment, NW fusion and encapsulation are two phenomena in competition. On one hand, the heat induces the fusion of the Cu NW junctions, which is expected to greatly increase the TCE conductivity by decreasing the contact resistance between the NWs. On the other hand, due to its low glass transition temperature (70 °C), the PET is softened during the thermal treatment. This causes the Cu NWs embedding inside the polymer substrate and, hence, a loss of conductivity. The challenge is thus to operate at a temperature where the fusion exceeds the encapsulation phenomenon, which will overall increase the TCE conductivity. It was found that fusion and encapsulation phenomena were dominating at 220 °C (TCE #1) and 230 °C (TCE #2), respectively. After a thermal treatment at 200 or 210 °C, no conductivity could be measured because there were still too many organic residues around the Cu NWs and they were not fused together. As a consequence, the contact resistance between the NWs was still very high. And after a thermal treatment at 240 or 250 °C, no conductivity could be measured because the encapsulation phenomenon was too important.

**Fig. 4:**
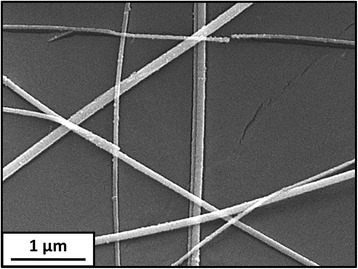
SEM image of the UV-treated TCE #3 (2 min) presenting unfused, non-encapsulated Cu NWs

The micrograph of Fig. 4 presents the high-magnification, top view of the TCE #3. The Cu NW surface appears clean and non-encapsulated, proving that the OM and PVP traces have been removed without softening and damaging the PET substrate. However, the NW junctions are not fused, which is likely due to a lower energy delivered by the UV lamp used in this study, compared to the high-power Xe flash lamps utilised by other authors [31, 37]. Besides, the Cu NW surface is slightly rough, which may be due to a beginning of oxidation. Reducing the distance between the UV lamp bulb and the TCE could allow to transmit a higher energy in a shorter time, and hence, to achieve the Cu NWs fusion while avoiding their oxidation.

**Fig. 5:**
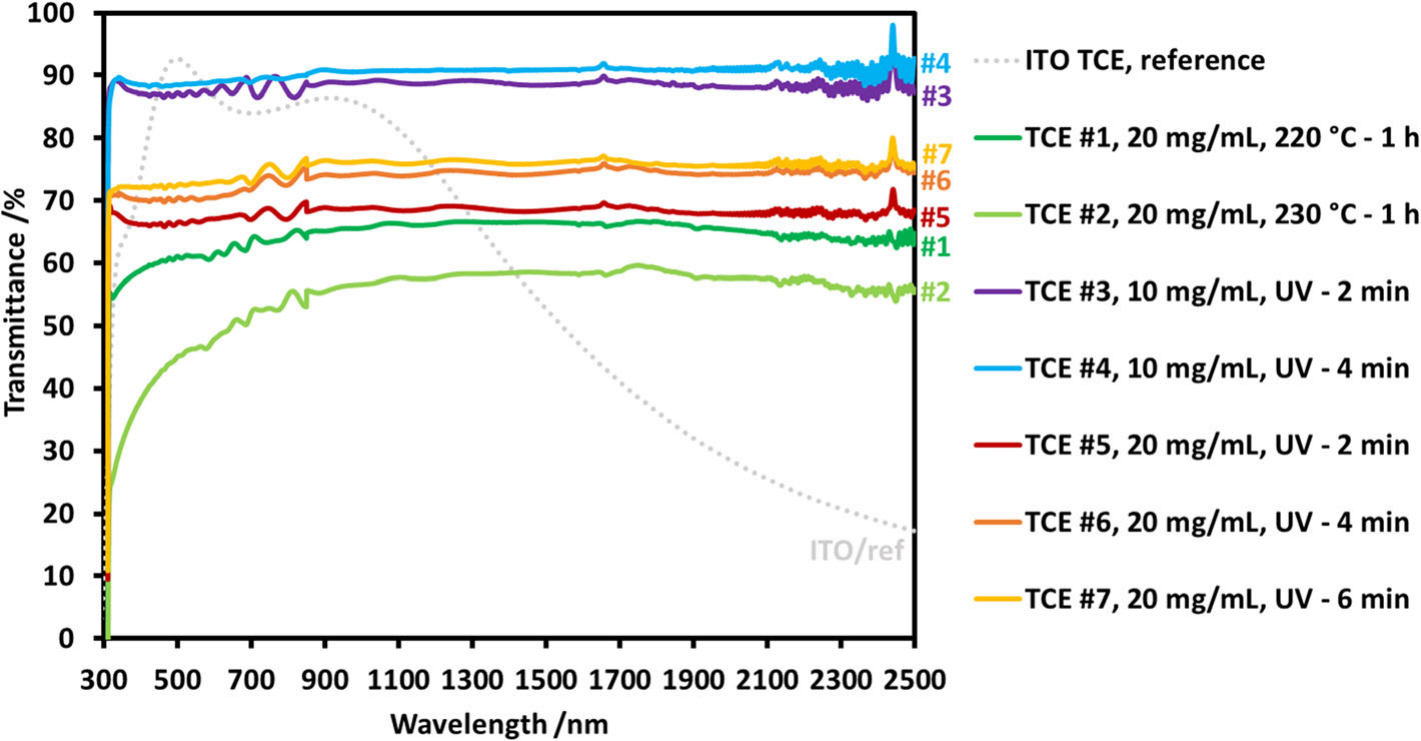
UV-Vis/NIR transmittance spectra between 300 and 2500 nm: they correspond to a commercial, glass-supported ITO TCE and to either thermally or UV-treated, PET-supported Cu NW TCEs

Table 1 shows the sheet resistance *R*s and the transmittance values *T*_550 nm_, *T*_350–750 nm_ (visible range) and *T*_750–2500 nm_ (IR range) of various Cu NW TCEs and of a commercial ITO TCE taken as reference. The transmittance spectra between 300 and 2500 nm are given in Fig. 5. For all the Cu NW TCEs, *T*_550 nm_ and *T*_350–750 nm_ are almost identical, proving that *T*_550 nm_ represents very well the average transmittance of a Cu NW TCE in the entire visible range. However, there is a difference of 6% between the two parameters for ITO/ref., which means that using *T*_550 nm_ instead of *T*_350–750 nm_ leads to an overestimated transparency in the visible range for that kind of transparent oxide.

An *R*s of 25 and 743 Ω sq^−1^, for a *T*_350–750 nm_ of 61 and 46% was measured for the TCEs #1 and #2, respectively. This confirms that the NW fusion prevails over their encapsulation inside the PET substrate at 220 °C, thus decreasing *R*s. The opposite occurs at 230 °C. Moreover, two reasons can explain the low *T*_350–750 nm_ values obtained for the TCEs #1 and #2, compared to 84% for ITO/ref. Firstly, the high concentration of Cu NWs in the ink (20 mg mL^−1^) lead to a high area fraction coverage. Secondly, the PET substrate was damaged during the thermal treatments.

As for the UV-treated TCEs, two main features were observed. Firstly, increasing the Cu NW concentration from 10 to 20 mg mL^−1^ decreased both the sheet resistance and transparency. After a 2-min UV treatment, *R*s decreased from 42 to 31 Ω sq^−1^ and the corresponding *T*_350–750 nm_, from 87 to 67%. After a 4-min UV treatment, *R*s decreased from 103 to 49 Ω sq^−1^ and the corresponding *T*_350–750 nm_, from 89 to 71%. This is in agreement with both theoretical and experimental results previously reported: increasing the area fraction covered by the NWs decreases both the sheet resistance and the transparency of a TCE [33–36]. Secondly, increasing the UV irradiation time increased significantly *R*s but only slightly the transparency. For instance, with an ink concentration of 20 mg mL^−1^, the TCEs #5 (2 min), #6 (4 min) and #7 (6 min) had an *R*s of 31, 49 and 236 Ω sq^−1^, with corresponding *T*_350–750 nm_ values of 67, 71 and 73%, respectively. And with an ink concentration of 10 mg mL^−1^ for the TCEs #3 and #4, *R*s increased from 42 to 103 Ω sq^−1^, with corresponding *T*_350–750 nm_ values of 87 and 89%. It is worth noting that those performances are quite similar to Wang et al.’s acid-treated TCEs (30 and 60 Ω sq^−1^ with a corresponding *T*_550 nm_ of 83 and 90%) [10]. They are also close to Mallikarjuna et al.’s flash lamp-treated TCEs (110 and 170 Ω sq^−1^ with a respective *T*_550 nm_ of 90 and 95%) [31]. The TCEs obtained from inks with concentrations of 10 and 20 mg mL^−1^ became non-conductive after UV treatments longer than 4 and 6 min, respectively. Regardless of the ink concentration, a low *R*s was obtained after 2 min of UV irradiation. This means that most of the organics were removed and that, despite the absence of fusion, the Cu NWs were in intimate contact. This was confirmed by the fact that the transparency after 2 min was very close to the one obtained after longer UV treatments. When the UV irradiation duration in ambient air increased, it is likely that oxidation occurred due to a temperature increase. The oxide layer on the NW surface grew thicker, thus increasing their contact resistance. However, it remained thin enough not to lower the transparency significantly. The quite stable transparency over the time range 2–6 min also means that the PET substrates were not degraded during the UV treatments. Consequently, only the high area fraction covered by the NWs on the TCEs #6 and #7 (20 mg mL^−1^ ink) was responsible for the *T*_350–750 nm_ values lower than for the ITO/ref. Indeed, with an ink concentration of 10 mg mL^−1^, *T*_350–750 nm_ for the TCEs #3 (87%) and #4 (89%) was slightly higher than for the ITO/ref. (84%).

Furthermore, it is worth noting that *T*_750–2500 nm_ is significantly higher for every Cu NW TCE than for the ITO/ref. (50%). This can be observed for the thermally treated TCEs #1 (65%) and #2 (57%), despite the PET substrates degradation. This is even more interesting for UV-treated TCEs, in particular #3 and #4, which have *T*_750–2500 nm_ values of 89 and 91%, respectively. This means that Cu NW TCEs suit applications such as IR imaging and sensing, electromagnetic shielding, telecommunications or IR solar cells much better than ITO, known to have a poor transmittance in the IR range [1, 7, 25, 29].

Finally, the comparison between the results obtained for thermally and UV-treated Cu NW TCEs highlights the advantages of the latter process. Quite close performances were measured for the thermally treated TCE #1 (25 Ω sq^−1^ with *T*_350–700 nm_ = 61%) and the UV-treated TCE #3 (31 Ω sq^−1^ with *T*_350–700 nm_ = 67%). However, the UV irradiation lasted 30 times less than the thermal treatment, and neither damaged the PET substrate nor required a controlled atmosphere. Furthermore, a UV treatment in ambient air is compatible with an industrial R2R process. Further work will consider a high-speed, low-cost, large-scale production using an R2R platform, a slot die, a UV lamp and an acid bath [38, 39]. Preliminary tests have already been realised at laboratory scale with a syringe pump injecting 15 mL h^−1^ of Cu NW ink in a slot die and a table moving a PET substrate at 10 mm s^−1^. So far, the results obtained on 2 × 5-cm^2^ coatings have suggested an optimal shim width of 100 μm and a slot die-substrate gap of 80 μm.

## Conclusions

High aspect ratio (length/diameter = 1000) Cu NWs were synthesised through a wet chemical, catalyst-assisted route. Then, they were used to fabricate TCEs on flexible PET substrates using the Meyer rod technique. A UV treatment and an acid bath were carried out to remove organic residues from the NW surface and obtain both low sheet resistance and high transparency. This method gave better results than a conventional thermal treatment, 30 times faster, and without needing a controlled atmosphere. Forty-two and 103 Ω sq^−1^, with corresponding *T*_350–750 nm_ of 87 and 89%, were the best performances obtained for UV-treated TCEs, which fits the requirements for flexible capacitive touchscreens. A very interesting result is that the transparency values of the Cu NW TCEs were maintained in the IR range, where the reference ITO TCE had a very low *T*_750–2500 nm_ of 50%. Hence, the Cu NW TCEs fabricated for this study are a very promising alternative to oxide TCEs for applications such as IR imaging and IR solar cells. Finally, the Cu NW ink and PET substrate, as well as the UV and acetic acid post-treatments used in this study, are compatible with an industrial, scalable, high-speed, low-cost, R2R process.

## Abbreviations

IPA: Isopropyl alcohol
IR: Infrared
ITO: Indium tin oxide
NP: Nanoparticle
NW: Nanowire
OM: Oleylamine
PET: Polyethylene terephthalate
PVP: Polyvinylpyrrolidone
R2R: Roll-to-roll
TCE: Transparent conductive electrode
UV: Ultra-violet
